# 956. Preliminary Investigation of Music Therapy's Impact on Medical Providers During Pediatric Burn Wound Care

**DOI:** 10.1093/jbcr/irag033.523

**Published:** 2026-04-06

**Authors:** Stephanie Epstein

**Affiliations:** Holtz Children's Hospital at University of Miami/Jackson Memorial Hospital (Miami Burn Center), Plantation, FL

## Abstract

**Introduction:**

Music therapy provides individualized, outcome-driven interventions targeted toward non-musical goals—in burn care, it can increase compliance in medical care; ^2^decrease pain, stress, and anxiety surrounding dressing changes; ^3^provide opportunities for emotional expression and coping; ^4^and improve compliance during physical and occupational therapies. While extensive literature cites the positive effects of music therapy on burn patients during procedures and in rehabilitation, little to no research explores the secondary effects and impact of music therapy on the medical providers during burn dressings and how that, in turn, can improve patient outcomes. Medical providers often report extensive anecdotal feedback regarding the positive impacts and effects of music therapy for them during burn dressings, including lower stress, increased and improved focus, and being able to complete the procedure more efficiently and effectively due to improved patient compliance. This preliminary investigation looks to explore the impact of music therapy on medical providers during pediatric burn wound care.

**Methods:**

To study this, the music therapist conducted semi-structured interviews with medical providers, including nurses, patient care technicians, and doctors. Questions asked providers to recall their experiences with music therapy during pediatric burn wound care (including debridement and dressing changes) with and without music therapy, including their ability to focus, their stress level, their perception of patient compliance and experience, and the overall experience for all individuals in the room (including the family when applicable).

**Results:**

Results showed a highly favorable perception of music therapy during burn wound care with respondents citing feeling more relaxed, less stress, better compliance by the patient, and a more favorable experience overall than when music therapy was not present. They also cited improvements in pain management, less crying, and faster recovery/return to baseline following the procedure.

**Conclusions:**

Preliminary results show the positive impact of music therapy on medical providers. Further research is needed to continue exploring the impact of music therapy on providers, including how the improvements for the medical providers then in turn further improves patient outcomes. Future research could include quantitative measurements (i.e., behavioral stress, vital signs, validated measurement tools) and biomarkers, such as cortisol levels.

**Applicability of Research to Practice:**

Findings of this research can be applied to pediatric burn patients in other hospitals as well as for adult burn patients, expanding the field of music therapy, providing more comprehensive patient care. The integration of music therapy into the interdisciplinary burn team addresses both patient distress and healthcare provider resilience, fostering a more supportive and comprehensive approach to burn care.

**Funding for the study:**

N/A.

